# What would you do first? A survey of treatment priorities for patients with hip-spine syndrome among spine and hip surgeons

**DOI:** 10.1186/s12891-026-09533-2

**Published:** 2026-01-23

**Authors:** Carolina Breuning, Xinggui Tian, Jens Goronzy, Klaus-Peter Günther, Uwe Platz, Franziska Beyer, Alexander Carl Disch, Paul F. Lachiewicz, Ning Liu, Stuart B. Goodman, Kirkham B. Wood, Stefan Zwingenberger

**Affiliations:** 1 ENT Department, Katharinenhospital Stuttgart, Kriegsbergstraße 60, Stuttgart, 70174 Germany; 2 Center for Orthopaedic, Trauma Surgery and Rehabilitation Medicine, University Medicine Greifswald, Fleischmannstraße 8, Greifswald, 17475 Germany; 3 Orthopedic Department, University Hospital Tübingen, Hoppe Seyler-Str. 3, Tübingen, 72076 Germany; 4 Spine Center, Schön Klinik München Harlaching, München, Germany; 5 University Center for Orthopaedic, Trauma and Plastic Surgery, University Hospital Carl Gustav Carus, , TU Dresden, Germany; 6 Department of Orthopaedic Surgery, Duke University School of Medicine, Durham, USA; 7 Department of Orthopaedic Surgery, Stanford University School of Medicine, Stanford, Redwood City, USA

**Keywords:** Hip-spine syndrome, Spine disease, Hip osteoarthritis, Surgical sequence, Treatment strategies, Decision-making

## Abstract

**Background and purpose:**

Hip-spine syndrome refers to the coexistence of hip and spinal pathologies, complicating diagnosis and surgical decision-making. This study investigated how German hip and spine surgeons prioritize surgical sequence in hip-spine syndrome and compared these patterns with those previously reported among U.S. surgeons.

**Methods:**

A cross-sectional survey containing five standardized case scenarios of concurrent hip osteoarthritis and typical degenerative spinal disorders was distributed to members of the German Society for Joint Replacement and the German Spine Society. Respondents included orthopaedic hip surgeons, orthopaedic spine surgeons (OSS), and neurosurgical spine surgeons (NSS). Quantitative data were analyzed using descriptive statistics and chi-square tests, and qualitative comments were examined using text-mining and thematic synthesis. Findings were compared on a point-by-point basis with the published results of the original U.S. study.

**Results:**

In general, German surgeons recommended spine-first treatment when neurological deficits such as myelopathy or neurogenic claudication were present, and hip-first treatment in cases without neurological deficits. Preferences differed significantly among specialties, especially between NSS and OSS. Compared with U.S. respondents, German surgeons were more likely to prioritize spine-first procedures in scenarios involving neurogenic claudication. Decision-making was primarily driven by symptom severity, spine-pelvis-hip biomechanics, and perceived procedural risk, rather than by surgeons’ years of experience.

**Conclusion:**

The preferred order of surgery for patients with hip-spine syndrome varies with neurological status, surgeon specialty, and geographic context. These results underscore the importance of interdisciplinary communication and individualized, patient-centered strategies when formal treatment guidelines are lacking.

**Trial registration:**

Not applicable.

**Supplementary Information:**

The online version contains supplementary material available at 10.1186/s12891-026-09533-2.

## Introduction

Hip-spine syndrome refers to the coexistence of hip and spinal disorders with overlapping symptoms, often complicating diagnostic evaluation and treatment decision-making [1]. Although the exact prevalence of hip-spine syndrome is not well established, degenerative disorders of the hip and spine are highly prevalent, particularly in aging populations, and reported estimates of coexisting pathology vary widely, ranging from approximately 21.2% to 61.5% [2]. In straightforward clinical situations, the dominant pathology usually determines the initial surgical target; however, in complex cases, deciding whether to treat the hip or spine first remains controversial. Despite increasing awareness of hip-spine syndrome, an evidence-based consensus on the optimal surgical sequence has yet to be established [1, 3]. Previous research has predominantly examined mechanical or outcome-based interactions between total hip arthroplasty (THA) performed for hip pathology and spinal fusion procedures performed for spinal pathology [4–8], while fewer studies have explored how clinicians prioritize operations in diverse real-world scenarios. Therefore, understanding scenario-specific clinical decision-making is of particular practical importance.

In clinical practice, joint and spine surgeons often encounter patients with hip-spine syndrome, most commonly presenting as hip osteoarthritis (OA) and degenerative lumbar spine disease. Decisions regarding which pathology to address first often vary between arthroplasty and spine specialists, partly reflecting differences in training background, operative priorities, and interpretation of symptom origin [9, 10]. Currently, no universal treatment guideline exists, highlighting the need for collaborative, interdisciplinary decision-making. In 2019, a research team from Stanford University surveyed U.S. hip surgeons (HS) and spine surgeons (SS) to evaluate surgical sequencing preferences across five standardized hip-spine syndrome scenarios [11]. Recognizing the distinct educational pathways and practice patterns in Germany [12], where both orthopaedic spine surgeons (OSS) and neurosurgical spine surgeons (NSS) are actively involved in spine care, the present study was designed to assess treatment preferences among German specialists. By directly comparing the German and U.S. survey results, this cross-national analysis aimed to explore cultural- and specialty-related differences in surgical decision-making and to inform more integrated, patient-centered management approaches for hip-spine syndrome.

## Methods

### Questionnaire design

The survey instrument was adapted from a previously published U.S. questionnaire originally designed by the Stanford University research team **(Figure ****S1****)** [11]. It presented five standardized clinical scenarios depicting patients with symptomatic hip OA and one of five common degenerative spinal disorders: (1) lumbar spinal stenosis with neurogenic claudication, (2) lumbar spondylolisthesis with leg pain, (3) single-level lumbar disc herniation causing leg weakness, (4) lumbar scoliosis accompanied by sagittal imbalance and back pain, and (5) thoracolumbar disc herniation with myelopathic signs. Each case included a concise clinical history, current symptoms, diagnostic details, and imaging description. Respondents were asked to select which operation they would perform first, THA or spinal surgery, and to briefly explain their reasoning in free-text comments. For respondents who selected THA as the initial procedure, an additional question addressed their preferred hip articulation (standard size head, large head > 32 mm, dual mobility implant, or constrained liner). The original English survey was translated into German by two senior orthopaedic surgeons and independently verified by a third senior physician. The finalized questionnaire was implemented through a web-based platform “LimeSurvey” (LimeSurvey GmbH, Hamburg, Germany). Reporting followed the “Checklist for Reporting Results of Internet E-Surveys (CHERRIES)” statement [13] **(Table ****S1****)**.

### Survey

This survey was distributed to 2,500 members of the “German Spine Society” (Deutsche Wirbelsäulengesellschaft, comprising both OSS and NSS) on March 26th, 2021, and 883 members of the “German Society for Joint Replacement” (Arbeitsgemeinschaft für Endoprothetik, mostly HS) on April 8th, 2021, *via* institutional mailing lists disseminated by the administrative offices of the respective societies. Inclusion criteria for participation included active membership in the respective societies and the completion of the survey, including all required questions. Respondents with incomplete answers or who did not meet the inclusion criteria were excluded from the analysis. Treatment-order preferences of different German surgical specialties across each scenario, as well as their association with years of surgical experience, were analyzed.

Free-text comments were analyzed with a text-mining workflow implemented in R (v4.0.4) using the *tidyverse* (v1.3.1), *tidytext* (v0.3.2), *widyr*, *igraph*, and *ggraph* packages to identify frequently used terms and co-occurrence patterns. Word frequencies were computed to provide an objective, data-driven overview of commonly used expressions across scenarios. After computing word frequencies, semantically similar terms were manually grouped in Excel, based on semantic similarity and clinical relevance, with data transferred using the *readxl* (v1.3.1) and *xlsx* (v0.6.5) packages. This manual step was intended as an interpretive synthesis rather than independent qualitative coding. Any discrepancies in thematic grouping were resolved through discussion until consensus was reached. The relative frequency of each term (density = specific word count / total word count) was calculated, and key interpretive themes were qualitatively synthesized from the most recurrent expressions.

Quantitative and qualitative results from the German survey were directly compared, item by item, with the published data from the U.S. survey conducted by Stanford University [11]. In Germany, participating surgeons included HS from the German Society for Joint Replacement and SS from the German Spine Society, encompassing both OSS and NSS subspecialists. In contrast, the U.S. study comprised HS from the North American Hip Society and SS from the Scoliosis Research Society, without distinction between OSS and NSS [11]. For consistency in the cross-national comparison, the German OSS and NSS categories were therefore combined into a single SS group.

### Statistical analysis

Descriptive statistics were expressed as means (± range) or proportions (%). Between-group differences in categorical variables were evaluated using the chi-square test. Statistical analyses were performed with SPSS version 20 (IBM Corp., Chicago, IL, USA). A p-value < 0.05 was considered statistically significant.

### Ethical considerations

The study protocol was reviewed and approved by the institutional ethics committee of the Technical University of Dresden (No. EK45012019). Participation was entirely voluntary and anonymous, with informed consent obtained electronically before survey initiation. The investigation was conducted in accordance with the ethical standards of the 1964 Declaration of Helsinki and its subsequent amendments.

## Results

### Participants

A total of 159 German surgeons participated in this study, including 62 HS (7.0%, 62/883) and 97 SS (3.9%, 97/2,500). For cross-national comparison, previously published data from a U.S. survey were referenced, which included 88 surgeons, comprising 51 HS (46.4%, 51/110) and 37 SS (36.6%, 37/101) [11]. The U.S. survey demonstrated a higher response rate than the German survey, likely reflecting differences in survey design, with the U.S. study employing a sample-based approach and the German survey targeting the full membership of the respective professional societies.

### Patterns of surgical sequence preference

Overall, German surgeons demonstrated a consistent pattern of treatment preference: a spine-first approach was favored in Scenarios 1, 3, and 5, while a hip-first approach predominated in Scenarios 2 and 4. Statistically significant differences were identified between NSS and OSS in Scenarios 1 and 2. When compared with the U.S. data, the preference patterns were broadly consistent for Scenario 2 (hip-first among both HS and SS) and Scenario 5 (spine-first among both HS and SS). In contrast, clear national differences were noted in Scenario 1, where U.S. surgeons more frequently chose a hip-first strategy, whereas German surgeons favored spine-first surgery. Additional variations were also seen in Scenarios 3 and 4, where U.S. HS showed fewer uniform preferences compared with the more consistent trends among German HS, SS, and U.S. SS. The detailed preference distributions are shown in Table 1.

**Table 1:** Preferred treatment sequence among German and U.S. Surgeons across five fictional clinical scenarios (U.S. Data from one published study by Stanford university [11])

	Germany				USA			
Scenario	Hip-first (%)	Spine-first (%)	No preference (%)	*p*	Hip-first (%)	Spine-first (%)	No preference (%)	*p*
**1** ^**a**^				0.24 (HS vs. SS)				0.47 (HS vs. SS)
HS	35.5	53.2	11.3		59	33	8	
SS	30.9	63.9	5.2		49	46	5	
NSS OSS	7.7 46.6	87.2 48.3	5.1 5.2	< 0.05* (NSS vs. OSS)	-			
**2** ^**b**^				0.10 (HS vs. SS)				1.00 (HS vs. SS)
HS	88.7	8.1	3.2		73	18	10	
SS	79.4	7.2	13.4		70	8	22	
NSS OSS	61.5 91.4	12.8 3.4	25.6 5.2	< 0.05* (NSS vs. OSS)	-			
**3** ^**c**^				0.36 (HS vs. SS)				< 0.05* (HS vs. SS)
HS	41.9	48.4	9.7		47	45	8	
SS	32	59.8	8.2		19	73	8	
NSS OSS	33.3 31	56.4 62.1	10.3 6.9	0.79 (NSS vs. OSS)	-			
**4** ^**d**^				0.95 (HS vs. SS)				< 0.05* (HS vs. SS)
HS	71	17.7	11.3		47	47	6	
SS	73.2	16.5	10.3		78	11	11	
NSS OSS	71.8 74.1	15.4 17.2	12.8 8.6	0.79 (NSS vs. OSS)	-			
**5** ^**e**^				0.13 (HS vs. SS)				0.07 (HS vs. SS)
HS	6.5	88.7	4.8		10	86	4	
SS	1	95.9	3.1		0	97	3	
NSS OSS	0 1.7	97.4 94.8	2.6 3.4	0.69 (NSS vs. OSS)	-			

“HS”, “SS”, “NSS”, and “OSS” refer to the hip surgeon, spine surgeon, neurosurgical spine surgeon, and orthopaedic spine surgeon, respectively. * *p* < 0.05

^a^ Lumbar canal stenosis with neurogenic claudication combined with osteoarthritis of the hip

^b^ Degenerative lumbar spondylolisthesis with radicular leg pain combined with osteoarthritis of the hip

^c^ Lumbar disc herniation with muscle weakness combined with osteoarthritis of the hip

^d^ Scoliosis with back pain combined with osteoarthritis of the hip

^e^ Thoracolumbar disc herniation with myelopathy combined with osteoarthritis of the hip

### Rationale for treatment-order decisions

In Scenario 1, German surgeons favoring the hip-first approach considered “hip” to be more “symptomatic”, contributing to increased lordosis of the spine and therefore recommending THA first. Those preferring a spine-first sequence viewed spinal “stenosis” as the more “symptomatic” condition. U.S. surgeons tended to favor hip-first surgery, reasoning that THA might improve spinopelvic biomechanics and relieve spinal symptoms, while those preferring spine-first emphasized that untreated neurogenic claudication may hinder the “recovery” of THA [11].

In Scenario 2, most German surgeons prioritized THA because of severe “symptomatic coxarthrosis” in the “hip”. They expected THA to improve mobility, restore spinal alignment, and facilitate rehabilitation for subsequent spinal surgery. Similarly, U.S. surgeons regarded hip pathology to be “serious” or “more severe” than radicular leg pain in the absence of neuromotor deficits and favored THA for its more predictable pain “relief” compared to “spinal fusion” [11].

In Scenario 3, some German surgeons opted for hip-first surgery, citing “femoral head necrosis” as an urgent “indication” for “hip”. Conversely, those who chose the spine-first approach considered neurological symptoms from the “disc” herniation, such as muscle weakness and paralysis, to be signs of impending nerve damage requiring urgent intervention. U.S. surgeons prioritized the spine-first approach with similar reasoning, emphasizing that muscle “weakness” is a significant “neurological deficit”, and that performing a “discectomy” for an extruded “disc” is a relatively straightforward procedure [11].

In Scenario 4, most German surgeons viewed “scoliosis” as a chronic condition with slower progression compared to the acute pain of hip pathology, thus favoring THA for faster pain relief and functional recovery. However, some prioritized spinal correction to restore “sagittal” imbalance before THA. Many US THA surgeons emphasized the importance of balancing the spine first to optimize the “position” of subsequent THA components, accommodating “changes” in spine-pelvic alignment post-spinal surgery. However, many U.S. HS surgeons selected a hip-first approach, assuming the patient’s “nerves” were intact and spinal surgery was not urgent, and SS considered THA as a less complex procedure with more “predictable” outcomes and quicker recovery than scoliosis surgery [11].

In Scenario 5, most German surgeons recommended spine-first treatment due to the presence of “myelopathy” and spinal cord “compression,” aiming to prevent irreversible neurological deficits. U.S. surgeons demonstrated comparable reasoning, considering “myelopathy” caused by “decompression of the spinal cord” a higher priority than managing hip OA [11].

**Fig. 1:**
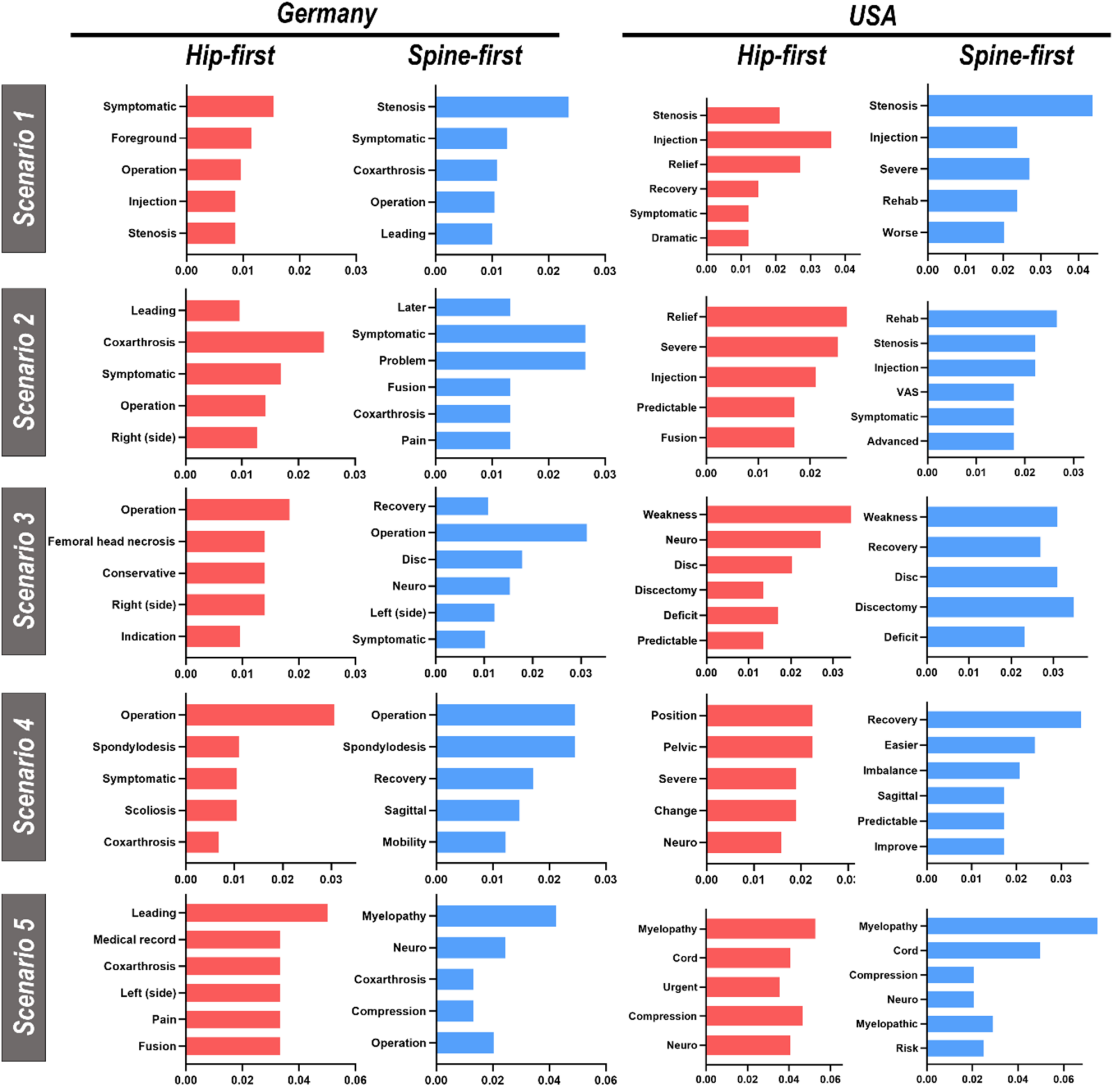
The most frequently used words in the surgeons’ comments for the five scenarios (*U.S. data are from one published study by Stanford University* [11])

The most frequently used terms from free-text comments are summarized in Fig. 1.

### Impact of surgical experience on treatment preference

**Fig. 2:**
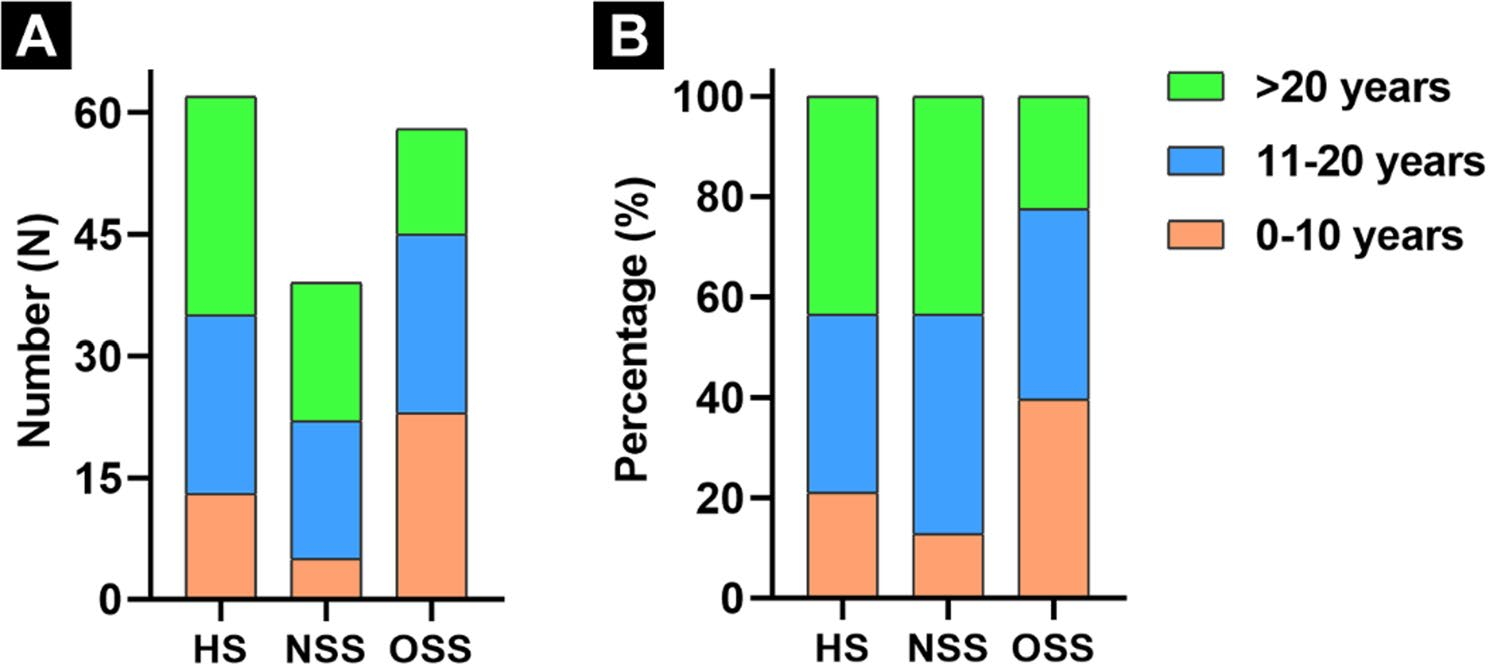
The number **(A)** and percentage **(B)** of participants in three different German specialties at different experience years after surgical training. “HS”, “NSS”, and “OSS” indicated the hip surgeon, neurosurgical spine surgeon, and orthopaedic spine surgeon, respectively

Overall, German respondents had a mean of 20.5 years of surgical experience (range 3–36) for HS, 20.1 years (range 2–34) for NSS, and 14.8 years (range 2–34) for OSS. Most HS reported 11–20 years of experience (35.5%), whereas NSS were evenly distributed between the 11–20 and > 20 years groups (both 43.6%). In contrast, OSS were more frequently represented in the 0–10 years group (39.7%) **(**Fig. 2**)**. In comparison, the U.S. survey reported longer average surgical experience, with a mean of 30.8 years (range 14–60) for HS and 23.4 years (range 5–34) for SS [11].

Experience level was not significantly associated with treatment preferences across any of the five clinical scenarios (*p* > 0.05). Overall preference patterns were consistent across experience groups, with a spine-first strategy favored in Scenarios 1, 3, and 5, and a hip-first strategy preferred in Scenarios 2 and 4. Nevertheless, descriptive trends were observed. In Scenario 1, increasing surgical experience was accompanied by a higher proportion of spine-first selections and a corresponding decrease in hip-first choices. In Scenario 2, hip-first preference appeared slightly lower among surgeons with more than 20 years of experience, while in Scenario 4, a modest increase in spine-first selection was noted with greater experience. In Scenario 5, hip-first selection was slightly more frequent among the most experienced surgeons, although the absolute proportion remained low **(**Table 2**)**.

**Table 2:** Treatment preferences across five fictional clinical scenarios among German surgeons, stratified by years of experience

Years of experience				
Scenario	Hip-first	Spine-first	No preference	*p*
**1** ^**a**^				0.44
0–10 years	43.9% (*n* = 18)	51.2% (*n* = 21)	4.9% (*n* = 2)	
11–20 years	29.5% (*n* = 18)	60.7% (*n* = 37)	9.8% (*n* = 6)	
> 20 years	28.1% (*n* = 16)	64.9% (*n* = 37)	7.0% (*n* = 4)	
**2** ^**b**^				0.32
0–10 years	87.8% (*n* = 36)	7.3% (*n* = 3)	4.9% (*n* = 2)	
11–20 years	86.9% (*n* = 53)	6.6% (*n* = 4)	6.6% (*n* = 4)	
> 20 years	75.4% (*n* = 43)	8.8% (*n* = 5)	15.8% (*n* = 9)	
**3** ^**c**^				0.90
0–10 years	34.1% (*n* = 14)	56.1% (*n* = 23)	9.8% (*n* = 4)	
11–20 years	39.3% (*n* = 24)	50.8% (*n* = 31)	9.8% (*n* = 6)	
> 20 years	33.3% (*n* = 19)	59.7% (*n* = 34)	7.0% (*n* = 4)	
**4** ^**d**^				0.62
0–10 years	68.3% (*n* = 28)	14.6% (*n* = 6)	17.1% (*n* = 7)	
11–20 years	75.4% (*n* = 46)	16.4% (*n* = 10)	8.2% (*n* = 5)	
> 20 years	71.9% (*n* = 41)	19.3% (*n* = 11)	8.8% (*n* = 5)	
**5** ^**e**^				0.37
0–10 years	0% (*n* = 0)	92.7% (*n* = 38)	7.3% (*n* = 3)	
11–20 years	3.3% (*n* = 2)	95.1% (*n* = 58)	1.6% (*n* = 1)	
> 20 years	5.3% (*n* = 3)	91.2% (*n* = 52)	3.5% (*n* = 2)	

^a^ Lumbar canal stenosis with neurogenic claudication combined with osteoarthritis of the hip

^b^ Degenerative lumbar spondylolisthesis with radicular leg pain combined with osteoarthritis of the hip

^c^ Lumbar disc herniation with muscle weakness combined with osteoarthritis of the hip

^d^ Scoliosis with back pain combined with osteoarthritis of the hip

^e^ Thoracolumbar disc herniation with myelopathy combined with osteoarthritis of the hip

### Choice of articulation type in THA

**Fig. 3:**
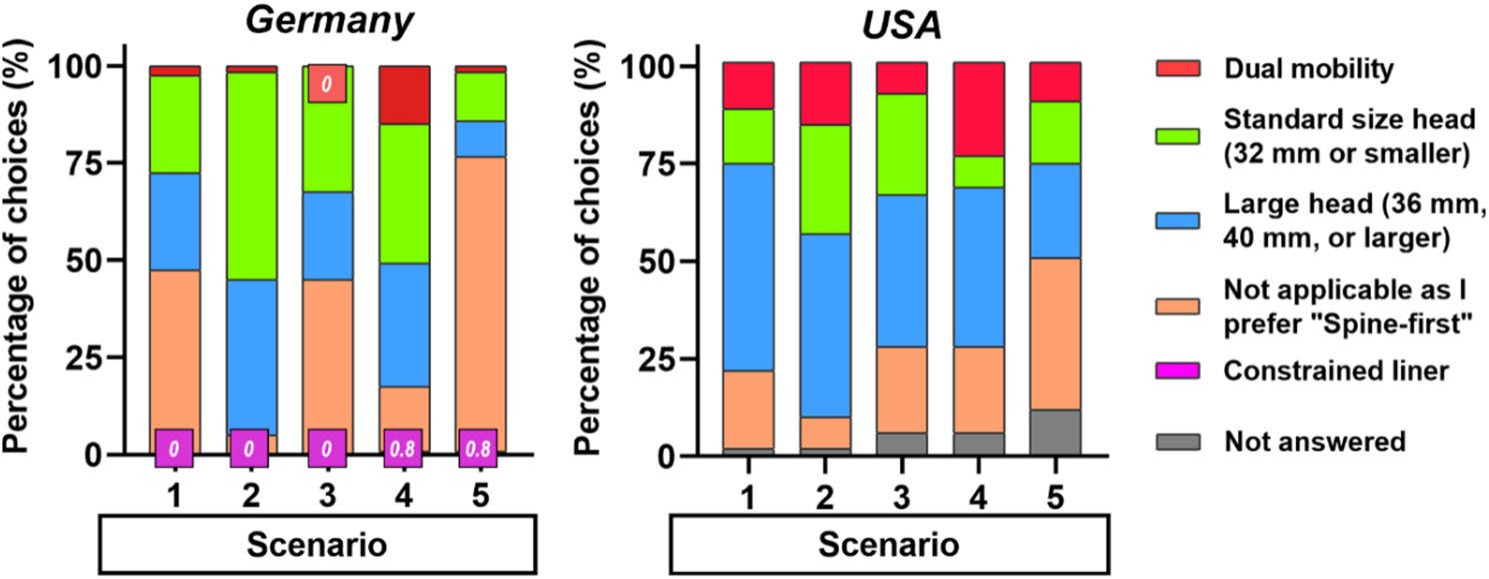
German and American surgeons’ choice of hip articulation type for different scenarios (*U.S. data are from one published study by Stanford University* [11]*)*. All participating German hip surgeons and orthopaedic spine surgeons answered this question, and none of the participating US surgeons chose a constrained liner option

Surgeons selecting a hip-first approach were asked to specify their preferred THA articulation type. NSS were excluded from this question due to scope-of-practice restrictions. Among German respondents, standard-size femoral heads were most frequently selected in Scenarios 2, 3, 4, and 5, while choices in Scenario 1 were nearly evenly divided between standard and large heads. In contrast, U.S. surgeons generally favored large-head components across all scenarios and reported a higher use of dual-mobility implants [11]. Notably, in Scenario 4, both German and U.S. surgeons showed a higher proportion of dual-mobility and large-head selections than in other scenarios, reflecting increased awareness of dislocation risk (Fig. 3).

## Discussion

Hip-spine syndrome remains a diagnostic and therapeutic challenge, particularly concerning the optimal sequence of hip and spine interventions [1, 3, 14]. The overlap of symptoms from hip OA and spine disorders frequently leads to diagnostic uncertainty and repeated cross-referrals between subspecialties [3]. This study analyzed how surgeons determine surgical sequence in cases of symptomatic hip OA with concomitant spinal disease, comparing patterns between German and U.S. surgeons.

### General patterns of preference

German respondents tended to favor a spine-first approach when neurological deficits were present, such as myelopathy, neurogenic claudication, or chronic neurological impairment, while a hip-first approach was chosen when no neurological deficit existed. U.S. surgeons showed a broadly similar trend. Specifically, both American HS and SS tended to prefer a spine-first approach in cases with myelopathy (Scenario 5), and American SS also favored spine-first in scenarios involving chronic neurological impairment (Scenario 3). Conversely, both American HS and SS favored a hip-first approach in conditions without neurological involvement in degenerative spondylolisthesis with leg pain (Scenario 2), and SS favored a hip-first approach in scoliosis with back pain (Scenario 4).

### Cross-national and professional differences

Clear contrasts emerged between national cohorts and surgical specialties in their preferred sequencing strategies. American surgeons, particularly HS, prioritized hip surgery more frequently despite chronic neurological deficits compared with their German counterparts. This distinction was most evident in cases of lumbar canal stenosis with neurogenic claudication (Scenario 1), where many American HS and SS selected a hip-first strategy, while German surgeons generally prioritized spine-first. A similar trend was observed in cases with chronic neurological impairment (Scenario 3), in which American HS more frequently opted for hip-first surgery. Within the American HS group, opinions diverged regarding scoliosis with back pain and concomitant hip OA (Scenario 4), reflecting ongoing debate about whether to restore spinopelvic balance or relieve hip-related symptoms first. Overall, these findings demonstrate that surgical sequencing decisions vary across clinical scenarios and reveal distinct cross-national differences in the weighting of neurological versus functional considerations.

When neurological deficits were present, a greater proportion of both American and German SS, particularly German NSS, chose a spine-first approach compared with HS (Scenarios 1, 3, 5). In contrast, HS showed a higher percentage preference for hip-first in most scenarios than SS, except in the case involving scoliosis with back pain (Scenario 4), where HS exhibited a lower preference for hip-first than SS. Significant differences were also observed between German NSS and OSS in Scenarios 1 and 2, as well as between U.S. SS and HS in Scenario 3. These patterns highlight how professional background and subspecialty focus shape surgical decision-making and underscore the importance of patient-centered, multidisciplinary coordination in managing hip-spine syndrome.

### Neurological symptoms shaping sequencing decisions

Across all cases, the extent of neurological deficits was the strongest factor influencing surgical order. Both cohorts prioritized spine-first treatment for myelopathy caused by thoracolumbar disc herniation (Scenario 5), consistent with previous views among HS and SS [9, 10, 15], and evidence supporting surgical intervention to optimize outcomes [16]. Neurogenic claudication, arising from nerve root compression or ischemia [17], was similarly viewed as requiring urgent decompression; therefore, the spine-first approach was favored in cases of lumbar canal stenosis with neurogenic claudication (Scenario 1). Muscle weakness, often linked to mechanical compression and inflammation of nerve roots and dorsal root ganglia from lumbar disc herniation [18], was another key indicator prompting spine-first intervention (Scenario 3). In contrast, when no neurological deficits were present (Scenario 2), most surgeons chose hip-first, citing hip symptoms as more likely to be severe than radicular leg pain in the absence of significant neurological deficits. For scoliosis (Scenario 4), a condition that is typically chronic and not associated with acute neurological deficits, most German surgeons, American SS, and half of the American HS regarded the spine pathology as nonurgent and chose to prioritize hip-first surgery.

### Biomechanical considerations

Hip-spine syndrome involves a complex interplay of the hip and spine pathology, symptoms, and biomechanics [15]. The interdependence between hip, spine, and pelvis biomechanics was another factor influencing sequencing decisions. Both degenerative spine and hip disease reduce segmental mobility, spinal fusion further restricts spinal motion, whereas THA restores hip flexibility and improves spinopelvic balance [15]. Accordingly, many surgeons, especially HS, advocated performing THA first in Scenarios 1 and 2 to improve spinopelvic biomechanics and relieve spinal symptoms, while some German SS favored spine-first correction to optimize acetabular positioning and gait in Scenario 1. In lumbar scoliosis with sagittal imbalance (Scenario 4), approximately half of American HS performed spinal correction first to optimize acetabular orientation for THA, consistent with previous guidance advocating deformity correction before THA to reduce dislocation risk [3]. Previous studies also recommend high-stability implants, such as large femoral heads or dual-mobility cups, for patients at greater risk of instability [4, 5, 19, 20]. This trend was evident among both cohorts, who more frequently selected dual-mobility components in Scenario 4, reflecting heightened awareness of postoperative instability. Sagittal imbalance may alter pelvic tilt, reducing functional acetabular coverage and increasing dislocation risk [21]. In such cases, achieving optimal cup position may require orientation outside the traditional “safe zone” [22]. American HS favored large femoral heads and dual-mobility constructs [11], whereas German surgeons typically chose standard-size heads.

### Procedural factors and surgical experience

Technical complexity and anticipated recovery also shaped treatment sequencing decisions. Although minimally invasive approaches have shortened recovery and reduced morbidity [23], spinal procedures generally remain technically more complex and are associated with higher complication rates compared with hip surgery [9]. In Scenario 3, some surgeons perceived discectomy as a relatively straightforward procedure and therefore favored a spine-first approach, whereas in Scenario 4, others considered THA to be less technically demanding and more predictable than scoliosis correction, supporting a hip-first strategy, particularly among U.S. surgeons. Despite the recognized benefits of minimally invasive techniques, their adoption is often associated with a substantial learning curve. Nevertheless, the present findings do not support a clear association between years of surgical experience and preferences for surgical sequencing.

### Clinical implications

Overall, the sequence of hip and spine surgery in hip-spine syndrome reflects a multifactorial decision process balancing neurological urgency, biomechanical considerations, and procedural complexity. German and U.S. surgeons applied similar principles but weighted them differently, reflecting distinctions in clinical training and health-system context. These results underscore the importance of a multidisciplinary and patient-specific approach when treating patients with combined hip and spinal pathology.

## Limitations

Several limitations of this study should be acknowledged. First, the survey design did not allow assessment of patient-reported or clinical outcomes associated with different surgical sequencing strategies; therefore, no conclusions can be drawn regarding the superiority of one approach over another. Second, although the total number of invited surgeons was substantial, the response rate was relatively low, introducing the potential for selection bias and limiting the generalizability of the findings. Third, the cross-sectional nature of the survey captures preferences at a single time point and does not permit evaluation of temporal changes in clinical decision-making. Fourth, the present analysis represents a bilateral comparison between German and U.S. surgeons and does not include perspectives from additional national or international professional societies, which may further limit the applicability of the results to global practice patterns. Finally, the survey focused exclusively on surgeon perspectives and did not incorporate patient preferences or shared decision-making considerations, which are increasingly recognized as important components in the management of hip-spine syndrome.

## Conclusion

This study provides novel insights into how surgeons prioritize the sequence of hip and spine surgery in patients with hip-spine syndrome. Treatment preferences varied according to surgeon specialty and geographic context and were primarily shaped by neurological severity, spine-pelvis-hip biomechanics, and perceived procedural complexity. German and U.S. surgeons demonstrated both shared decision-making principles and distinct cross-national differences, likely reflecting variations in training background and healthcare system structures. Across both cohorts, the presence of neurological deficits was consistently associated with a preference for a spine-first approach, whereas the absence of such deficits more often supported a hip-first strategy to improve mobility and spinopelvic alignment. These findings highlight the importance of interdisciplinary communication and individualized, patient-centered decision-making when managing patients with hip-spine syndrome.

## Supplementary Information

Supplementary Material 1.

## Data Availability

All data generated or analyzed during this study are included in this article and its supplementary materials.

## Abbreviations

OA: Osteoarthritis
THA: Total hip arthroplasty
HS: Hip surgeon
SS: Spine surgeon
OSS: Orthopaedic spine surgeon
NSS: Neurosurgical spine surgeon
